# A retroviral late-domain promotes cell-surface expression, extracellular vesicle formation, and immunogenicity of a trimeric HIV-1 Env antigen

**DOI:** 10.21203/rs.3.rs-11098456/v1

**Published:** 2026-10-12

**Authors:** Hao Li, Naomi Bronkema, Assem Ashimova, Lizhou Zhang, Nickolas Skamangas, Maria Ericsson, Gogce C. Crynen, Hyeryun Choe, Charles C. Bailey, Brian D. Quinlan, Michael Farzan

## Abstract

The immunogenicity of membrane-associated antigens can be enhanced by addition of cytoplasmic-tail elements that promote release of extracellular vesicles (EVs). Here we explore whether these elements enhance humoral responses to SOSIP-TM, a stabilized form of the HIV-1 envelope glycoprotein (Env). We compared modifications designed to promote EV release by engaging the cellular Endosomal Sorting Complex Required for Transport (ESCRT). These modifications include viral late domains and the previously described EPM-EABR, which combines an endocytosis prevention motif (EPM) with the ESCRT- and ALG-2-interacting protein X-binding region (EABR) of the cellular protein CEP55. We observed that p2, an 18 amino-acid ESCRT-associated feline immunodeficiency virus (FIV) late domain, promoted higher SOSIP-TM expression and a stronger total antibody response than other EV-enhancing modifications. p2 also elicited significantly higher neutralizing responses to a quaternary Env epitope than did EPM-EABR. FIV p2 thus promotes humoral responses to Env-based transmembrane antigens more effectively than other ESCRT-engaging elements.

### Introduction

The unprecedented success of mRNA vaccines against COVID-19 suggests that the mRNA-lipid nanoparticle (mRNA-LNP) platform can be used for many applications (1). One key advantage of mRNA-LNP over protein-based subunit vaccines is that membrane-anchored proteins can readily serve as immunogens. Membrane association markedly enhances the immunogenicity of viral entry proteins like the SARS-CoV-2 spike (S) protein (2, 3). Membrane association also allows introduction of immunogenicity-enhancing elements into the cytoplasmic tail of an antigen without disrupting antigen structure or exposing irrelevant epitopes. Hoffmann et al. have introduced two functional domains into the cytoplasmic tail of the S protein (4), an ESCRT- and ALIX-binding region (EABR) of the cellular protein CEP55, which promotes EV formation, and an endocytosis prevention motif (EPM) derived from the cytoplasmic region of FcγRII-B1, which promotes plasma membrane localization. Together, these domains promote release of extracellular vesicles incorporating the S-protein antigen, and improve humoral responses to this antigen, potentially increasing the level of antigen presented by follicular dendritic cells in the germinal centers of vaccinated mice. These studies have been extended to vaccines for respiratory syncytial virus and to mosaic antigens presenting the SARS-CoV-2 receptor-binding domain (5–8).

This strategy relies on the cellular ESCRT-complex machinery, which promotes dissociation of daughter cells during cytokinesis, neck formation and scission of vesicles and viruses budding from the plasma membrane or into the lumen of a multivesicular body (9–11). Two well characterized proteins, TSG101 and ALIX, help mediate these processes. TSG101 is a core component of an early step in vesicle release and is a key component of the ESCRT-I heterotetramer. Many enveloped viruses exploit the ESCRT system to promote their release through short late-domain sequences that include a characteristic P(T/S)AP domain recognized by TSG101. ALIX functions as an alternative or accessory adapter to recruit proteins mediating the penultimate step of vesicle formation in which downstream ESCRT-III proteins, including CHMP48, polymerize at the neck of the budding vesicle or virus, driving membrane scission (12). ALIX can function independently from TSG01, but it also interacts with TSG101 to coordinate nucleation of ESCRT-III proteins. Notably, the EABR binds and recruits both TSG101 and ALIX, as does the p9 late domain of equine infectious anemia virus (EIAV) and the p6 late domain of HIV-1 (13). In contrast, the p2 late domain of the feline immunodeficiency virus (FIV) interacts with TSG101, but its association with ALIX has not been detected (14).

Here we describe a cytoplasmic tail element that more effectively promotes the immunogenicity of stabilized HIV-1 envelope glycoprotein (Env) trimers. We compared cell-surface expression, EV release, and immunogenicity of trimeric Env antigens modified with cellular and viral late domains, with or without an EPM. We observed that the late-domain element of FIV gag protein, FIV p2, promotes cell-membrane expression like the EPM and vesicle release comparable to other ESCRT-associated proteins including EABR. This compact domain elicited a more potent antibody response to stabilized Env antigens in mice than any other late domain evaluated, with or without an EPM. Thus, inclusion of a cytoplasmic FIV p2 domain can improve the immunogenicity of membrane-associated Env antigens.

### Results

## EPM and FIV p2 enhance cell-surface expression of SOSIP-TM

Hoffmann et al. have shown that an EABR late-domain motif derived from the human protein CEP55 can, when combined with an endocytosis prevention motif (EPM), enhance the immunogenicity of a SARS-CoV-2 S-protein-derived transmembrane antigen (4). We sought to determine if the same general approach might also enhance humoral responses to analogous HIV-1 Env trimer antigens. We accordingly generated nine variants of a previously described SOSIP-TM variant (16055.ConM-v8.1ds; Fig. 1A-B) (15). This SOSIP-TM includes only the first 10 residues of the Env cytoplasmic tail, a truncation that improves Env surface expression relative to variants with complete cytoplasmic regions. These SOSIP-TM variants differ in their ESCRT-recruiting domains, which are the EABR, the p9 domain of EIAV, and the p2 domain of FIV. Several variants also include the EPM domain used by Hoffmann et al. Two variants included a foldon domain (Fd), derived from the bacteriophage T4 fibritin. This Fd domain promotes trimerization of these cytoplasmic tails, and were included to more closely emulate the trimeric arrangement of late domains in a retroviral capsid. Each individual domain was separated by a GGGS or GGSGG linker.

These variants were first characterized for their ability to express on the cell surface. Expi293F cells were transected with plasmids expressing SOSIP-TM variants H1 through H9. Cells were stained with the VRC26.25 and PGT151. VRC26.26 is an HIV-1 broadly neutralizing antibody (bnAb) that recognizes the quaternary apex epitope of Env and is used to monitor the expression of correctly folded SOSIP-TM trimers (16). PGT151 binds an epitope formed at the interface between the gp120 and gp41 domains of Env and is used to monitor total SOSIP-TM expression (Fig. 1C-D) (17). Three variants fused to the EPM (H5), FIV p2 (H7), or both (H8) expressed on the cell surface more efficiently than unmodified SOSIP-TM (H1). Thus, both FIV p2 and EPM increased surface expression of a SOSIP-TM antigen, but the combination of the two afforded no additional benefit. Of note, the EPM-EABR H3 variant, which is most similar to the S-protein trimer variants characterized by Hoffmann et al., expressed less efficiently on the cell surface, as did the same variant lacking an EPM (H9), implicating the EABR element in the lower expression of these variants. The EIAV late domain combined with the EPM domain (H2) approximated expression of unmodified SOSIP-TM (H1). All Fd domain-containing variants(H4, H6) expressed less efficiently than unmodified SOSIP-TM, consistent with the possibility that this foldon domain trimerizes across SOSIP-TM trimers. We conclude that both EPM and p2 promote cell-surface expression, whereas EABR markedly impairs this expression even when combined with an EPM.

**FIV-p2 promotes antibody responses to a SOSIP-TM antigen.**

To evaluate the ability of these antigens to raise anti-SOSIP-TM antibodies, we divided these SOSIP variants into two groups, with H1 and H5 common to both. The first group included H6, H7, H9, and the second included H2, H3, and H8. BALB/c mice were immunized twice, at an interval of 30 days, with 1 µg mRNA-LNP encoding each SOSIP-TM variant (Fig. 2A, **Supplementary Fig. 1**). Serum samples were collected just before the second immunization on day 30 (Fig. 2B, E), and at days 45 (Fig. 2C, F) and 60 (Fig. 2D-G) and characterized by ELISA for binding to a soluble SOSIP identical to the ectodomain of the SOSIP-TM immunogen. Each variant was compared with the unmodified H1 SOSIP-TM. Only mice immunized with H7 generated significantly higher titers of anti-SOSIP-TM antibodies than those immunized with H1, and this difference was evident at all three time points. In general, the significance and magnitude of these differences receded over time. Although not statistically significant, and H3 and H9, both containing EABR, consistently generated higher anti-SOSIP titers than H1, although lower than H7. We conclude that FIV p2 domain can enhance humoral responses to a SOSIP-TM antigen, and that its contribution to immunogenicity is more pronounced in these studies after a first immunization than at later time points.

To understand how the cytoplasmic domains of these SOSIP-TM variants contributed to their immunogenicities, we selected five variants (H1, H3, H5, H7, H9) for further characterization. Total cellular expression was assessed by Western blotting of lysates from cells transduced to express these SOSIP-TM variants (Fig. 3A). In most instances, total SOSIP-TM expression correlated with cell-surface expression. For example, the H3 variant, with low cell-surface expression relative to the H1 control, also retained less protein within the cell, whereas H5 and H7, with EPM or FIV p2 cytoplasmic domains, respectively, was present at markedly higher levels than H1. H9 levels in whole cell lysates were greater than its cell-surface expression would indicate, suggesting that the EABR domain alone (without an EPM) promoted removal of SOSIP-TM from the cell surface. We also investigated EV formation and SOSIP-TM incorporation into EVs through Western blotting of EVs isolated from cell cuture supernatants (Fig. 3B-C). As expected, unmodified SOSIP-TM (H1) and the EPM-only variant (H5) did not increase EV formation over background, as indicated by the tetraspanin EV markers C9, CD63, and CD81. In contrast, markedly greater EV formation was observed with H3, H7, and H9. Consistent with previous reports, TSG101 incorporated comparably into all EV, but ALIX was enriched only in EABR-containing variants (18). Incorporation of SOSIP-TM corresponded to tetraspanin incorporation, shown in Fig. 3C, implying the EABR domain present in H3 and H9 and the FIV p2 domain in H7 promoted EV formation in the vicinity of these SOSIP-TMs. Among these, EPM-EABR (H3) promoted the highest levels of EV formation, as indicated by Western blotting with the anti-gp41 antibody 4E10. Because Fig. 3C presents a non-reducing gel, a gp41 band was not expected, and we infer that the engineered SOSIP disulfide bonds that link the gp120 and gp41 Env subunits were less stable or never formed in some H3 protomers. Incorporation levels of uncleaved or disulfide-linked SOSIP-TM were similar across the three variants. To better characterize differences in SOSIP-TM incorporation of H1, H3 and H7 into EVs, EVs released from SOSIP-TM-transfected cells were captured by beads through their CD9, CD63 and CD81 incorporated into these vesicles (Fig. 3D). SOSIP-TM levels on captured EVs were quantified by flow cytometry using fluorescently labeled VRC26.25 and PGT151. All three capture beads and both antibodies showed that EV incorporation of H3 and H7 exceeded that of the H1 control, and that more H3 was incorporated than H7. Two populations of H3 were recognized by the antibody VRC26.25, which distinguishes between closed and open Env structures, but a single population was recognized by the interface antibody PGT151, suggesting that two distinct populations of EV expressed H3 on their surface. Finally, we examined cells transfected to express H1, H3, and H7 by staining with immunogold-labeled PGT151 and transmission electron microscopy (Fig. 3E). Abundant irregular vesicles or filopodia rich in PGT151-reactive H7 SOSIP-TMs were observed in proximity to or associated with H7-transfected cells, whereas far fewer such structures were observed in cells transfected with H3. In H1-transfected cells, nearly all immunogold label was associated with the plasma membrane rather than with vesicular membranes. To determine if the high levels of protein expression associated with the p2 domain were unique to SOSIP-TM, we appended this domain to CCR5, CD9, influenza A virus M2, and Epstein-Barr virus LMP1. Total and cell surface expression of these diverse proteins (**Supplementary Fig. S1**) were also markedly enhanced by the p2 domain, suggesting that this domain could enhance expression and retention of many transmembrane proteins.

**FIV-p2 promotes neutralizing antibody responses to a quaternary HIV-1 Env epitope.**

It has been observed in multiple settings that wild-type mice do not raise potent neutralizing responses to HIV-1 Env-based antigens, including SOSIP-TM (19, 20). To monitor the impact of the EPM/EABR and FIV-p2 modifications of SOSIP-TM on neutralizing responses to the quaternary apex epitope of HIV V2-glycan/apex bnAbs, C57BL/6 mice were engrafted with B cells isolated from the spleens of congenic mice and engineered to include the heavy chain CDR3 (HCDR3) region of the neutralizing apex bnAb VRC26.25 (Fig. 4A) (19). B-cell receptors engineered in this way recognized the quaternary apex epitope of HIV-1 Env and only bind properly assembled, closed-conformation SOSIP-TM antigens (21). Excepting their common human-derived HCDR3 sequence, these engineered B-cell receptors include the original mouse variable and constant heavy-chains regions as well as the original mouse light chain. Engineered B cells also largely retain the underlying diversity of the mouse repertoire. We have previously demonstrated that these engineered cells proliferate, affinity mature, and generate neutralizing antibody responses targeting the SOSIP-TM apex epitope (19, 20). We observed here that ELISA-measured humoral responses to soluble SOSIP trimers were markedly greater in mice engrafted with engineered B cells and immunized with H1 or H7 relative to identically vaccinated but unengrafted control mice seven days after the first immunization (Fig. 4B), indicating that most of this initial response could be attributed to the HCDR3-engineered cells. Consistent with this inference, serum from engrafted mice receiving this priming vaccination generated stronger neutralizing activity in a TZM-bl neutralization assay (Fig. 4C). H7-immunized mice generated stronger responses than those immunized with H1 or H3. Surprisingly sera from H1 control mice surpassed that of H3-immunized mice at this early time point. These significant differences largely persisted, but were of smaller magnitude, at day 22 after the first boost (Fig. 4D and 4E). The differences between H3- and H7-immunized mice may suggest that cell-surface expression of H7 promotes an early plasma cell response.

To further explore differences among these antigens, we immunized mice a third time at day 29, collected serum at day 36 and harvested spleens at day 39. Differences between H3 and H7 sera in binding and neutralization persisted at day 36 (Fig. 5A-B). Splenic B cells were also analyzed. No significant differences among these groups were observed in the number of B cells bearing germinal center markers (GL7+, CD38-; Fig. 5C). However significantly greater numbers of CD45.1-positive engrafted cells were present in the spleens of H3-immunized mice at day 39 (Fig. 5D). Higher numbers of CD45.1-donor cells from these mice expressed germinal cell markers (Fig. 5E) and were recognized by a soluble SOSIP protein identical to the ectodomains the SOSIP-TM immunogens (Fig. 5F), although these latter differences did not reach significance. However, a higher proportion of antigen-reactive cells were found in H7-immunized mice in both total or CD45.2-positive host mouse cells were monitored (Fig. 5G-H). These data imply that by day 39 after three immunizations, the H3 antigen promoted higher germinal cell retention of engrafted cells, whereas H7-promoted stronger humoral responses from unmodified host mouse B cells. Collectively these data are consistent with a model in which a strong early plasma response in H7-immunized mice generated high serum levels of apex-recognizing neutralizing antibodies, and that epitope masking suppresses germinal center retention of donor B cells targeting this epitope. In contrast, H3-immunized mice did not generate an early competing response, and their germinal-center retention of apex-binding antibodies after three vaccinations was = s accordingly greater. Alternatively, higher EV generation in cells expressing H3 may increase the amount of GC-bound antigen, whereas higher expression of H7 in APC may enhance plasma cell formation.

**FIV p2 enhances both prime and boost immunizations.** Because serum differences between H3- and H7-immunized mice were less significant after boosting, and because in previous reports EPM-EABR showed greater differences from unmodified antigen at later time points, we compared H3 and H7 separately in prime and boost immunizations (Fig. 6A). These studies also used a lower mRNA dose (0.5 µg) and included a new variant, H10, with two tandem FIV p2 domains. As expected, after a single prime immunization, sera generated from H7-immunized mice bound the SOSIP antigen more efficiently than H1- and H3-immunized mice at 15 and 29 days post-immunization (Fig. 6B-C), although only comparisons between H7 and H3 were statistically significant. H10 was not significantly different from H7 despite its higher expression and EV formation (**Supplementary Fig. 3**). To see if H3 performed differently in the context of a boost, each group of 10 mice was then divided in two subgroups (n = 5), arranged so that the geometric mean serum binding, as measured by ELISA, was equivalent in both subgroups and distributions were similar (**Supplementary Fig. 4**). Each subgroup was boosted with either H3 or H7. In all four comparisons, mice boosted with H7 generated significantly higher binding sera than those boosted with H3 (Fig. 6D-E), and the magnitude of the gain from day 29 was also significantly greater (Fig. 6F). We conclude that H7 generates significantly more binding antisera to SOSIP-TM than H3 after both prime and boost vaccinations.

### Discussion

Despite the breakthrough success of mRNA vaccines, further improvements to their efficacies are needed to lower their required doses and thus many of their side-effects. More effective vaccines could elicit immune responses against hard-to-neutralize targets including cancer epitopes, malaria antigens, or HIV-1. In the specific case of HIV-1, a leading vaccine approach employs a stepwise series of immunizations intended to induce B cells expressing rare precursor antibodies to generate protective antisera. The viability of this approach decreases rapidly with the number of necessary immunizations, and therefore advances that increase the efficacy of each step can bring us closer to an effective and practical vaccine. Hoffmann et al. have forwarded one approach for enhancing the humoral response to a SARS-CoV-2 S-protein-based antigen, by introducing elements into its cytoplasmic domain (4). They have shown that combining two cytoplasmic-tail elements, one that promotes cell-surface expression (EPM) and one that facilitates extracellular vesicle formation from mRNA-LNP transfected cells (EABR), results markedly higher EV production and stronger humoral responses. This approach was subsequently validated with the respiratory syncytial virus fusion protein and in the context of mosaic SARS-CoV-2 receptor binding-domain antigens (5, 7).

Here we extend this approach to a stabilized HIV-1 Env-based antigen, SOSIP-TM (15). In contrast to the SARS-CoV-2 S protein, we found that immunogenicity improvements contributed by EPM-EABR to SOSIP-TM were small, and that EPM and EABR each contributed to these modest effects. It is unclear why EPM-EABR underperformed relative to previous reports with other viral antigens, but we note that the EABR region participates directly in dimerization of CEP55 (18). A propensity to dimerize within or across membrane-bound trimers may be more disruptive to a trimeric Env-based antigen like SOSIP-TM compared with, for example, the also trimeric SARS-CoV-2 S-protein antigen. Alternatively, the virtual absence of H3 on transfected cells, and its high levels on EVs, suggest that this EPM-EABR-modified antigen will be presented differently to the immune system than the unmodified H1 control or the FIV p2-modified H7, and that S proteins and Env antigens have distinct requirements for engaging a naïve mouse B-cell repertoire. The differing SOSIP-TM species associated with EV generated from H3- and H7-transfected cells may also contribute to qualitatively different responses to these antigens.

We also evaluated the viral late domains of EIAV and FIV in this setting and observed that the 18-amino acid FIV p2 late domain elicited significantly greater antibody titers than unmodified SOSIP-TM or any other combination of cytoplasmic elements tested here. These observations correlated with another property unique to p2. Specifically, p2 mediated markedly higher surface and total expression of SOSIP-TM in the LNP-transduced cell, while still promoting efficient EV formation (summarized in Fig. 7). We further demonstrated that the total amount of p2-modified antigen in the cell is greater, suggesting that FIV p2 may prevent lysosomal degradation of SOSIP-TM. It is unclear whether greater amount of protein in the cell results in higher or lower amounts of MHC class II presentation of SOSIP-TM-derived peptides. At first pass, higher cellular antigen content should increase antigen presentation through MHC II. However, elements that prevent endocytosis present in EPM and p2 may also prevent access to the MHC class II-containing compartment (MIIC) where antigen processing for MHC II is thought to occur. However, the very low surface and total expression of EPM-EABR-modified SOSIP-TM antigen suggests that class II presentation of its peptides on LNP-transduced antigen-presenting cells will be lower than with unmodified or p2-modified antigens. Markedly higher levels of SOSIP-TM on the LNP-transfected cell surface may also facilitate direct engagement of B cells with antigen-expressing APCs outside the germinal center (22–24). Efficient extrafollicular presentation of SOSIP-TM may promote early development of antibody-secreting plasma cells, improving serum neutralization and broadening the response, but suppressing further affinity maturation of BCR recognizing the same epitope (25). Our data are consistent with this framework. For example, the response to H7 is higher relative to H1 or H3 after a single prime immunization, implying an early plasma-cell response. However, H7-immunized mice also generated higher antigen-binding serum after one or two boosts regardless of the initial priming antigen.

Interestingly, after three immunizations, H3 promoted strong germinal center activity against a single epitope from engineered B cell, although H7 generated stronger antigen-binding responses to the entire antigen. A straightforward explanation for this observation is that the original epitope was masked by antibody secreted from plasma cells in response to earlier immunizations with H7. Regardless, our data make clear that p2-modified SOSIP-TM promotes stronger humoral responses than does the unmodified antigen or SOSIP-TM modified with EPM-EABR.

The molecular mechanisms underlying the cellular differences between antigens modified by FIV p2 and EABR also remain incompletely defined. Both domains interact with the ESCRT protein TSG101, but p2 does not interact with ALIX (26), whereas the association of ALIX with the EABR domain of CEP55 is well documented (18). This difference could be quantitative, and the ALIX-binding domain in EABR may be too efficient in promoting EV release, leaving little protein in the transfected cells and limiting an initial priming response. In contrast, the relative inefficiency of p2 may result incomplete scission of nascent EV, as TEM evidence suggests, facilitating recognition of LNP-transfected cells at the interface of the B and T-cell zones of the lymph node. Alternatively, ALIX may also direct EV to the multivesicular body, where a fraction of EV is transported to the lysosome and digested. In addition to differences in ALIX engagement, EABR also differs from p2 in the manner it engages TSG101. Viral late domains like the PSAP motif of p2 bind directly to the TSG101 to a cleft of its UEV domain and are ubiquitin dependent. CEP55 binds TSG101 at its proline rich region distal from and independent of TSG101’s ubiquitin modifications. These differences may account for the greater efficiency of EV formation observed with EABR as well as its more rapid degradation within the cell. Consistent with this possibility, over-expression of TSG101 can accelerate lysosomal degradation of CEP55 (27). Finally, FIV p2 may have intrinsic EPM-like activity in addition to its late domain function. Inspection of p2 sequences downstream of its PSAP motif suggests a mechanism shared between p2 and EPM, but further studies will be necessary to confirm this relationship. Regardless of mechanism, this property of p2 is general, because it promotes cell-surface and total expression of transmembrane proteins with a range of functions and topologies, including viral antigens such as Epstein-Barr virus LMP1, and influenza A virus M2, as well human targets of therapeutic antibodies including CCR5 and CD9. It is currently a challenge to raise conformational antibodies against labile transmembrane proteins (28), and p2 may help overcome this difficulty.

In summary, we have identified an 18-amino acid sequence of FIV p2 that can enhance total and cell-surface expression of HIV-1 Env antigens similarly to EPM (48 amino-acids), promote EV formation more efficiently than unmodified SOSIP-TM, and generate more potent antibody responses antibodies than EPM, EABR (58 amino acids) or EPM-EABR (111 amino acids). Further investigation of its ability to promote antibody responses to other transmembrane antigens, especially whose target epitope is poorly preserved in the FDC network, may extend its utility beyond HIV-1 Env-based trimers.

### Materials and Methods

## Mice

All animal experiments were performed in compliance with institutional guidelines and approved IACUC protocols at Boston Children’s Hospital (Protocols 00002782 and 00002783) and The Broad Institute of MIT and Harvard (Protocol 0351-12-22). For studies without B-cell engraftment in Fig. 2, 6–8-week-old female BALB/c mice (Jackson Laboratory, strain 000651) were used for immunizations, with 5–7 animals per group. 1 µg of mRNA encoding SOSIP antigens was administered to mice as mRNA-LNP via intramuscular (IM) injection on days 1 and 31. Serum samples were collected on days 30, 45, and 60. For studies in Fig. 6, BALB/c mice were also used except that 0.5 µg of mRNA was administered and blood was collected at days 15, 29, and 47. For studies using engineered B cells, nine to 12 week-old female CD45.1-positive mice (B6.SJL-Ptprca Pepcb/BoyJ, strain 002014) from Jackson Laboratories were used as a source of splenic B cells. Age- and sex-matched CD45.2-positive C57BL/6J strain mice (Jackson Laboratories, strain 000664) strains were used as host mice for B cell transplantation and immunizations. The neutralizing response to vaccine candidates was evaluated as previously described (15). Splenic B cells isolated from $CD45.1^+$ donor mice were edited by replacing the mouse HCDR3 with that of VRC26.25 in a background of mouse VH1-family encoding variable chains. These edited B cells were then engrafted into $CD45.2^+$ recipient mice via retro-orbital injection as previously described. 24 hours after engraftment of edited B-cells, 2 µg of mRNAs was administered into the recipient mice as mRNA-LNP, followed by two more doses on days 15 and 29. Immunized mouse sera were collected on days 8, 22 and 36. Identically vaccinated mice that were not engrafted with engineered B cells served as negative controls. All procedures were performed on mice anesthetized with isoflurane.

### DNA construction

Codon-optimized DNA sequences encoding a previously described HIV-1 SOSIP-TM construct, 16055-ConM-v8.1ds (15) fused with cytosolic elements were cloned into a mammalian expression vector under the control of a CMV promoter. All constructs contain the SOSIP ectodomain followed by the gp41 transmembrane domain (TMD) from ConM SOSIP Env. A series of endosomal sorting and/or ESCRT-binding elements were fused to the C-terminal of this SOSIP-TM. Coding sequences of SOSIP-TM and its fusion variants were flanked by 5’- and 3’-UTRs (AGCAAATAAGAGAGAAAAGAAGAGTAAGAAGAAATATAAGACCCCGGCGCCGCCACC and GCTGGAGCCTCGGTGGCCTAGCTTCTTGCCCCTTGGGCCTCCCCCCAGCCCCTCCTCCCCTTCCTGCACCCGTACCCCCGTGGTCTTTGAATAAAGTCTGAGTGGGCGG[illegible] respectively) and adapted from Moderna’s mRNA-1273 vaccine construct. Following the CMV promoter, a T7 promoter was also inserted upstream but adjacent to the 5’UTR and a 101-nucleotide polyA tail was also introduced after 3’UTR region to function as an in vitro transcription template. Amino-acid sequences of all C-terminal fusion element sequences are described in Fig. 1.

## mRNA synthesis and purification

High-fidelity DNA polymerase (Invitrogen, 12368010) was used to amplify the target DNA template for mRNA in vitro transcription (IVT). A 101-nucleotides long poly(A) stretch was introduced to the template during PCR using a reverse primer that contains the poly(dT) stretch at its 5’ end. Amplified IVT templates were precipitated using isopropanol and further purified by using a PCR cleanup kit (Machery-Nagel, 740609). mRNAs were synthesized by using a protocol modified from MEGAscript® T7 Transcription Kit (Invitrogen, AMB13345) (29). Briefly, N1-methylpseudouridine-5'-triphosphate (TriLink, N-1081) was used to replace uridine-5'-triphosphate (UTP) and CleanCap AG (3' OMe), a cap 1 analog (TriLink, N-7413), was used to co-transcriptionally cap the mRNAs. After a 4-hour IVT reaction, DNA templates were digested via Turbo DNase and then precipitated by adding 2.5M lithium chloride to the reaction. mRNA pallets were washed with 70% ethanol in nuclease-free and low endotoxin water (Cat# W3440, Teknova). To remove dsRNAs generated during the IVT and reduce its immunogenicity, mRNAs were then dissolved and underwent cellulose purification (30). mRNAs were further purified using Monarch Spin RNA Cleanup Kit (New England Biolabs, T2050S). mRNA purity and concentrations were determined by NanoDrop, and verified by 1.25% agarose gel electrophoresis (Lonza, 54922).

### Production of mRNA-lipid nanoparticle (LNP)

mRNAs were formulated into lipid nanoparticles by rapidly mixing mRNAs in aqueous phase with lipids in ethanol using NanoAssemblr™ Ignite™ nanoparticle formulation systems (Cytiva). To prepare the two phases for formulation, first mRNAs were resuspended in 25 mM sodium acetate buffer (pH5.0) at 0.1 mg/mL. Separately, four lipids, SM-102 (MedChemExpress, HY-134541) DSPC (Avanti Polar Lipids, 850365P), cholesterol (Sigma-Aldrich, C8667) and 14:0 PEG2000 PE (Avanti Polar Lipids, 880150P), were dissolved in ethanol with a corresponding molar ratio of 50:10:38.5:1.5. Formulations were mixed at an N/P ratio of 6, a flow rate ratio of 1.67:1 (aqueous phase:organic phase), and a total flow rate of 12 mL/min. After formulation, mRNA-LNPs were dialyzed three times against cold $Ca^{2+}$ and $Mg^{2+}$ free DPBS immediately by using dialysis cassettes with 10K MWCO (ThermoScientific, 66810). mRNA-LNP encapsulation efficiency and concentrations of encapsulated mRNA were measured before being aliquoted and frozen in 10% sucrose in -80 degree Celsius. Quant-iT™ RiboGreen assay (Life Technologies, R11490,) was used to measure the concentration of encapsulated mRNA and mRNA encapsulation efficiency following manufacturer's instruction. LNP particle's diameter was measured in DPBS at 25°C by using multi-angle Dynamic Light Scattering (DLS) (Malvern Panalytical, Zetasizer Ultra). LNP size distribution, or polydispersity index, was measured by using back scatter DLS in Zetasizer Ultra.

### Cell-surface expression of vaccine constructs

200 ng plasmid DNA encoding indicated SOSIP-TM variant (H1-H9) was transfected into around 3 million Expi293F cells in 1 mL media using FectoPRO transfection reagent (Polyplus, 101000014). Alternatively, 750 ng of SOSIP-TM- or 125 ng SARS-CoV-2 S protein-encoding LNP were transfected into the same number of Expi293F cells. Transfected Expi293F cells were incubated at 37°C, 8% CO2 with 200 rpm agitation. 48 h post-transfection, 100 µL transfected Expi293F cells were transfer to a 96 well plate followed by flow cytometry assay.First, cells were stained in FACS buffer consisting of PBS supplemented with 10% FBS and 0.1% sodium azide. Cells were then pelleted at 500 × g for 4 min at 4°C in 96-well V-bottom plates and resuspended in staining solution containing fluorophore-conjugated antibodies VRC26.25-FITC and PGT151-APC. for 30 min at 4°C protected from light. After incubation, cells were washed twice with ice-cold FACS buffer, resuspended in 80 µL FACS buffer, and kept at 4°C for flow cytometric acquisition.

## Western blots

8.5 µg mRNA-LNPs encoding SOSIP-TM (16055-ConM-v8.1ds-TM) variants H1, H3, H5, H7 and H9 were transfected into 17 mL of 3 million per mL Expi293F cells. 24 h post-transfection, supernatants and transfected cells were separated and harvested via centrifugation at 3000 x g at 4°C. Supernatants containing extracellular vesicles (EVs) were subjected to further purification following the protocol (Qiagen, 76064). EVs were resuspended in the same volume of elution buffer, and 10% sucrose was added before they were aliquoted and stored at -80°C. Transfected cell pellets were also stored at -80°C before use. The presence of SOSIP-TM on the purified EVs and in the transfected cells was detected by Western blot analysis. The same volumes of purified EVs were added into SDS-PAGE loading buffer under non-reducing conditions before protein electrophoresis into SDS page gels (Thermo Fisher Scientific, NP0321BOX). The gel was transferred to a PVDF membrane (MilliporeSigma, IPVH00010) using semi-dry transfer method under 25 V for 30 min and SOSIP antigens were detected using 1 µg/mL 4E10 antibody (BEI source, ARP-10091), a human bnAb targeting the membrane-proximal region of HIV-1 Env. The blot was imaged using a horseradish peroxidase (HRP)-conjugated goat antihuman IgG1 (0.4mg/mL) diluted in 1:10,000 (SouthernBiotech, 2014-05). Western blot analysis on cell lysates transfected with SOSIP-TM variants was conducted in the same conditions.

## Protein production

Plasmids expressing antibodies of PGT151 and CAP256-VRC26.25 were transfected into Expi293F cells using FectoPRO reagent (Polyplus, 101000014) with ratio of 1:1.5 of light chain and heavy chains, respectively. Supernatants were harvested 3–4 days post transfection, and antibodies were captured with protein A columns (Cytiva, 17549851) and eluted with IgG Elution Buffer, pH 2.0 (Thermo Scientific, 21028). Eluted antibodies and elution buffer were then exchanged to PBS and concentrated by centrifugal filtration. SOSIP proteins were produced in house and at Duke Human Vaccine Institute. Specifically, protein was produced in Expi293F cells by transient transfection using FectoPRO reagent. 4–5 days post transfection, supernatants containing SOSIPs were harvested and purified using PGT145 affinity column chromatography. SOSIPs were further purified by FPLC size exclusion chromatography using Superdex 200 Increase 10/300 GL column (Cytiva, 28990944) and stored in 10 mM Tris HCl and 500 mM NaCl (pH 8.0) buffer at -80°C. In preliminary studies, SOSIP protein was produced under the direction of Dr. Kathy Yarborough in the Duke Human Vaccine Institute Research Protein Production Facility which received funding support from the Collaboration for AIDS Vaccine Research Bill and Melinda Gates Foundation (OPP1066832).

## B-cell engineering

Engineering of primary mouse B cells so they express heavy-chain CDR3 regions of apex antibodies has been previously described (15). Specifically, whole spleens from 9–12 week old CD45.1-positive C57BL/6 donor mice were pulverized and mechanically crushed on the inner top of 70 mm cell strainers in RPMI 1640 medium (Thermo Fisher Scientific, 61870127) with 2% FBS (Thermo Fisher Scientific, 26140-079). After red blood cell lysis in a $NH_4Cl$ solution (BD Biosciences, 555899) at room temperature for 3 minutes, B cells were neutralized with Ca2+/Mg2 + free PBS with 0.5% BSA (Sigma-Aldrich, A1470) and

5 mM EDTA and then isolated using mouse B cell purification kit (Miltenyi Biotec, 130-090-862) and LS columns (Miltenyi Biotec, 130-042-401). Before electroporation, B cells were activated for 36–42 hours in RPMI 1640 medium with 10% FBS, 100 mM Non-Essential Amino Acids (NEAA, Thermo Fisher Scientific, 11140050), 1 mM sodium pyruvate (Thermo Fisher Scientific, 11360070), 10 mM HEPES (Thermo Fisher Scientific, 15630080), 55 mM 2-Mercaptoethanol (Thermo Fisher Scientific, 21985023), 100 units/mL penicillin and 100 mg/mL streptomycin (Thermo Fisher Scientific, 15140163), and either (1) 4 mg/ml anti-mouse CD180 antibody (BD Biosciences, 562191), (2) 50 mg/ml LPS (Sigma-Aldrich, L2880), or (3) 10 mg/ml LPS and 10 ng/ml mouse IL-4 (PeproTech Inc, 214 – 14). After activation, B cells were harvested and washed twice with Ca2+/Mg2 + free PBS at room temperature. For each 100 ml of electroporation reaction using the nucleocuvette vessels (Lonza, V4XP-4024), approximately 5 million cells were suspended in 74 ml of P4 Primary Cell solution (Lonza, VSOP-4096). In parallel, 3.12 ml of PBS, 1.26 ml of 1M NaCl, 1.12 ml of 250 mM Mb2Cas12a (produced in house), and 4.5 ml of 100 mM gRNA (5'-UAAUUUCUACUGUUUGUAGAUCUUGACCCCAGUAGUCCAUAGCA-3') were mixed and incubated at room temperature for 15 min for the RNP complex formation. These 10 ml of RNP were then incubated with 16 ml of 100 mM single strand DNA donor for 3 minutes at room temperature. The above 26 ml mixture was then mixed with the 74 ml of suspended B cells and transferred to the nucleocuvette vessels for electroporation in the Lonza 4D nucleofector under the DI-100 program. For larger 1 ml scale electroporations using the Nucleocuvette Cartridge (Lonza, V4LN-7002), reactions were scaled up by a factor of 10 for cell suspension, RNP, and donors. After electroporation, cells were rested for 10 minutes in nucleocuvette vessels or cartridges and then transferred to preheated activation medium without penicillin-streptomycin or LPS, which were added one hour later.

## ELISA

ELISA was performed to evaluate vaccine-induced antibody response to the ectodomain of the SOSIP-TM antigen in vaccinated mouse sera. 96-well plates (Corning, 3690) were coated with 0.5-1.0 μg/mL 16055-ConM-v8.1ds SOSIP trimers in DPBS overnight at 4°C. ELISA plates were then washed with PBST (PBS, 0.05% Tween-20, pH 7.4) before they were blocked with 3% BSA (w/v) (Sigma-Aldrich, A7030) in PBST for at least 1 hour at room temperature. After blocking, sera from immunized mice were first diluted 1: 30 or 1:100, and then serially diluted 2- to 6-fold in PBST buffer containing 3% BSA and transferred to plates. After 1 hour incubation at room temperature, plates were washed with a plate washer. Plates were then incubated with 50 μL of Fab-specific, goat anti-Mouse IgG labeled with peroxidase (Sigma-Aldrich, A9917) with the dilution for 1:4000 in 3% BSA PBS solution for 30 minutes at room temperature. After incubation with the secondary antibody, plates were washed 5 times and 50 μL of Ultra TMB-ELISA substrate (Thermo Scientific, 34028) was added. The reaction was developed for 10 mins before it was halted with 50 μL of TMB Stop solution (Southern Biotech, 0412-01). Absorbance at 450 nm (OD450) was measured with a Victor Nivo plate reader. Area under the curve (AUC) values were calculated from ELISA binding curves using GraphPad Prism version 11.0.0. $OD_{450}$ values were plotted against log-transformed serum dilution, and AUC was determined using the trapezoidal rule across the dilution range tested.

## Pseudovirus production and neutralization assays

HIV-1 pseudoviruses were produced by transient transfection of HEK293T cells with the HIV-1 backbone plasmid pNL4-3.Luc.R-E- (BEI Resources, HRP-3418) and an HIV-1 16055 Env expression plasmid (BEI Resources, ARP-11502) at a 5:1 mass ratio. HEK293T cells were seeded in T225 flasks and transfected at ~ 60–75% confluency with ~ 48 μg total plasmid DNA using PEIpro transfection reagent (Polyplus, 101000017). Transfected cells were incubated at 37°C, and culture supernatant containing pseudoviruses were harvested 48 h post-transfection. Supernatants were clarified by centrifugation at 4,000 x g for 10 min, followed by filtration through a 0.45 μm membrane filter and were aliquoted and stored at -80°C until use. TZM-bl neutralization assays were performed as previously described (31). Briefly, serially diluted mouse sera in 96-well plates were incubated with pseudotyped viruses at 37°C for 1-1.5 hour. TZM-bl cells were then added to the wells at 10,000 cells/well. Cells were incubated at 37°C for 48 h before they were lysed in wells and subjected to firefly luciferase assays. Luciferase activity was detected using the Britelite Plus reagent (Revvity, 6066761). Luminescence was measured using a Victor Nivo plate reader (PerkinElmer). Neutralization titers ($ID_{50}$) were calculated as the serum dilution causing a 50% infection in relative luminescence units (RLU) compared with virus-only control wells. Neutralization curves were fitted by using Log(inhibitor) vs normalized response -- Variable slope (four parameters) nonlinear fit model in GraphPad Prism version 11.0.0.

## Germinal Center Staining

Splenocytes were isolated from mice and after red blood cell lysis were stained with a panel against important B cell markers. IgM, IgG B cells were identified as the CD45R+ (Biolegend 103224), CD3- (Biolegend 100240), IgD- (Biolegend 405740) population. CD38- (Biolegend 165616) GL7+ (Biolegend 144612). B cells were identified as germinal center B cells. We distinguished between engrafted and non-engrafted B cells with staining for CD45.1+ (BD Biosciences 757856) B cells as our engrafted population. APC-labeled ConMv8.1ds SOSIP was also included to identify antigen-positive B cells. Splenocytes were resuspended with B-cell Isolation Buffer (BCIB; StemCell Technologies 20144) containing mouse Fc blocker (1:50) (Miltenyi Biotec, 130-092-575). Splenocytes were resuspended at a concentration of 0.25 million cells per ul. Per 20 million cells, splenocytes received 1 ul αCD38, αIgD, 2 ul α-GL7, α-CD45.1, α-CD3, SOSIP, α-CD45R, and 8 ul BCIB for a total of 20 ul. After 15 minutes on ice, cells were washed once and resuspended with BCIB containing 7AAD to identify viable cells and then analyzed on the CytoFlex S flow cytometer.

## Statistical analyses

For ANOVA and other parametric tests, model adequacy was assessed by examining residual-versus-predicted plots and the distribution of residuals for each outcome. Residuals were checked if they were normally distributed, with no heteroscedasticity or systematic patterns were observed. If residuals indicated non normal distribution or heteroscedasticity, the data was transformed. Statistical tests were two-sided, and $P < 0.05$ was considered statistically significant.

Differences in ELISA outcomes across the five vaccine groups were evaluated independently for each production batch (Batch 2 and Batch 3) at three distinct time points: Day 30, Day 45, and Day 60. For each batch and time point, a one-way analysis of variance (ANOVA) was performed in JMP Pro v19 (SAS Institute Inc., Cary, NC, USA) to test for main effects of vaccine type. When the overall ANOVA model was statistically significant, post hoc comparisons against the control group (H1) were conducted using Dunnett's test to control multiple comparisons, reporting adjusted 95% confidence intervals and adjusted *p*-values.

For flow cytometric studies, each cell subpopulation count was modeled beta-binomial regression in Generalized Regression in JMP Pro v19 (SAS Institute Inc., Cary, NC, USA) to account for the excess variability where vaccine group was the fixed effect. Beta-binomial model was evaluated using residual diagnostics and goodness-of-fit measures. When the main test p value < 0.05, pairwise comparisons among the three vaccine groups were performed using or Dunnett's test to account for the multiple comparisons.

Post-boost ELISA AUC values were analyzed using an analysis of covariance (ANCOVA) in JMP Pro v19 (SAS Institute Inc., Cary, NC, USA). The initial model included prime vaccine construct, boost vaccine construct, and their interaction as fixed categorical effects, with prime-stage ELISA AUC included as a continuous covariate. This model evaluated differences in post-boost responses while adjusting for variation in each animal's prime-stage response. Model assumptions were assessed using residual-versus-predicted plots and the distribution of residuals. The residuals were approximately normally distributed, and no substantial heteroscedasticity or systematic pattern was observed; therefore, untransformed AUC values were analyzed. Adjusted group means were estimated as least-squares means. Because the boost factor contained only two levels, its overall F-test provided the direct comparison between boost constructs, and no additional post hoc test was required.

### Declarations

**Author Contributions:** HL, CCB, BDQ, and MF designed experiments. HL, NB, AA, ME conducted experiments, with critical guidance and assistance from LZ, HC, and CCB. HL, and BDQ. HL, CB, BDQ, and MF conceived the project. HL and GCC performed statistical analyses. HL and MF wrote the manuscript.

**Competing Interest Statement:** HL, BDQ, and MF have filed patent disclosures describing the use of the FIV p2 domain to promote vaccine efficacy. The remaining authors declare they have no competing interests.

Acknowledgments: The authors would like to thank Drs. Wenhui He and Tianling Ou for their insights during the development of these studies, and Margaret Hellwarth in the Scripps Research Institute International Service Office for her invaluable assistance and advice. The following NIH awards are also gratefully acknowledged: R01-AI174270 (MF); R01-AI174277 (HC, MF); R01-DA056771 (MF); R01 AI186793 (MF).

### Figures

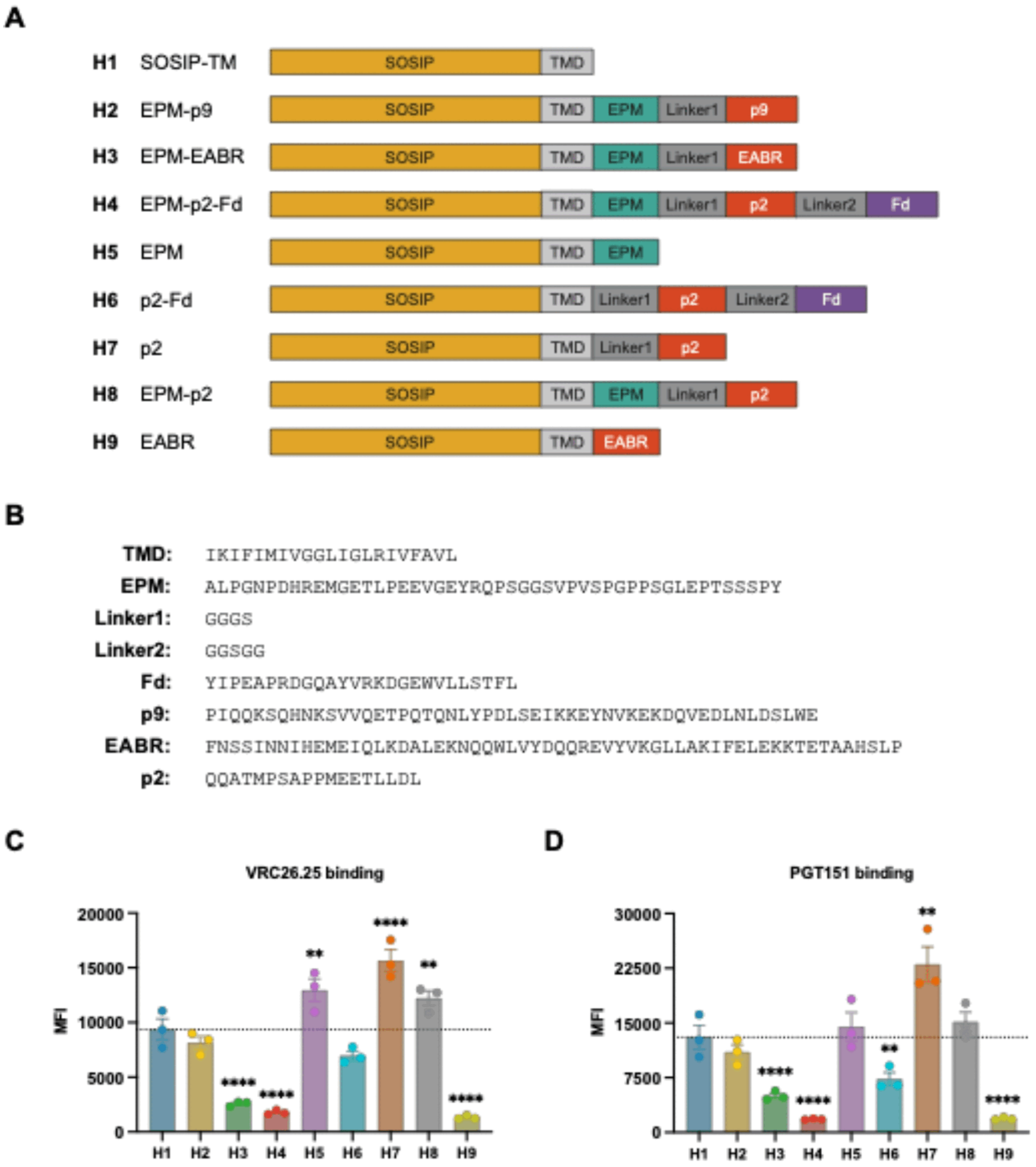

Figure 1

**Schematic of SOSIP-TM constructs with various cytoplasmic tail elements regulating cell-surface expression and EV formation.**

**A** A representation of characterized SOSIP-TM variants. SOSIP-TM (H1) is a previously described stabilized Env trimer (SOSIP v8.1ds) based on a consensus of HIV-1 group M (ConM) with a V1V2 region derived from the clade C 16055 isolate, and a ConM transmembrane (TMD) with a truncated 10 amino-acid cytoplasmic domain (deltaCT, not represented) (19). Each of the remaining variants (H2-H9) are fused at the SOSIP-TM carboxy terminus with elements regulating cell-surface expression and EV formation. Thus EPM (endocytosis prevention motif) is derived form from a murine Fc gamma receptor FcgRII-B1 cytoplasmic tail; p9 is the late-domain from the equine anemia virus gag protein, EABR is domain from the cell protein CEP55, described in Hoffmann et al.; p2 is the late domain of the feline immunodeficiency virus (FIV) gag protein. In addition, Linker1 and Linker2 indicate four and five amino-acid glycine-rich linkers, respectively, and Fd is the foldon trimerizing domain from bacteriophage T4 fibritin. **B** Amino-acid sequences of the cytosolic elements defined in panel A. **C-D** Expi293F cells transfected with DNA encoding the indicated SOSIP-TM variants were stained with the apex bnAb VRC26.25 (C), recognizing conformationally intact SOSIP-TM, or the interface antibody PGT151 (D), which recognizes a wider range of SOSIP-TM configurations. Averages of geometric mean fluorescence intensities (MFI) of three biological replicates are presented. Horizontal dotted lines indicate geometric mean MFI of H1 control Geometric mean fluorescence intensity (MFI) from VRC26.25 and PGT151 binding across SOSIP-TM constructs were compared using a one-way ANOVA and when significant, followed by Dunnett's test against the control group H1 SOSIP-TM variant. (**P < 0.05; **P < 0.01; ***P < 0.001; ***P < 0.0001*).

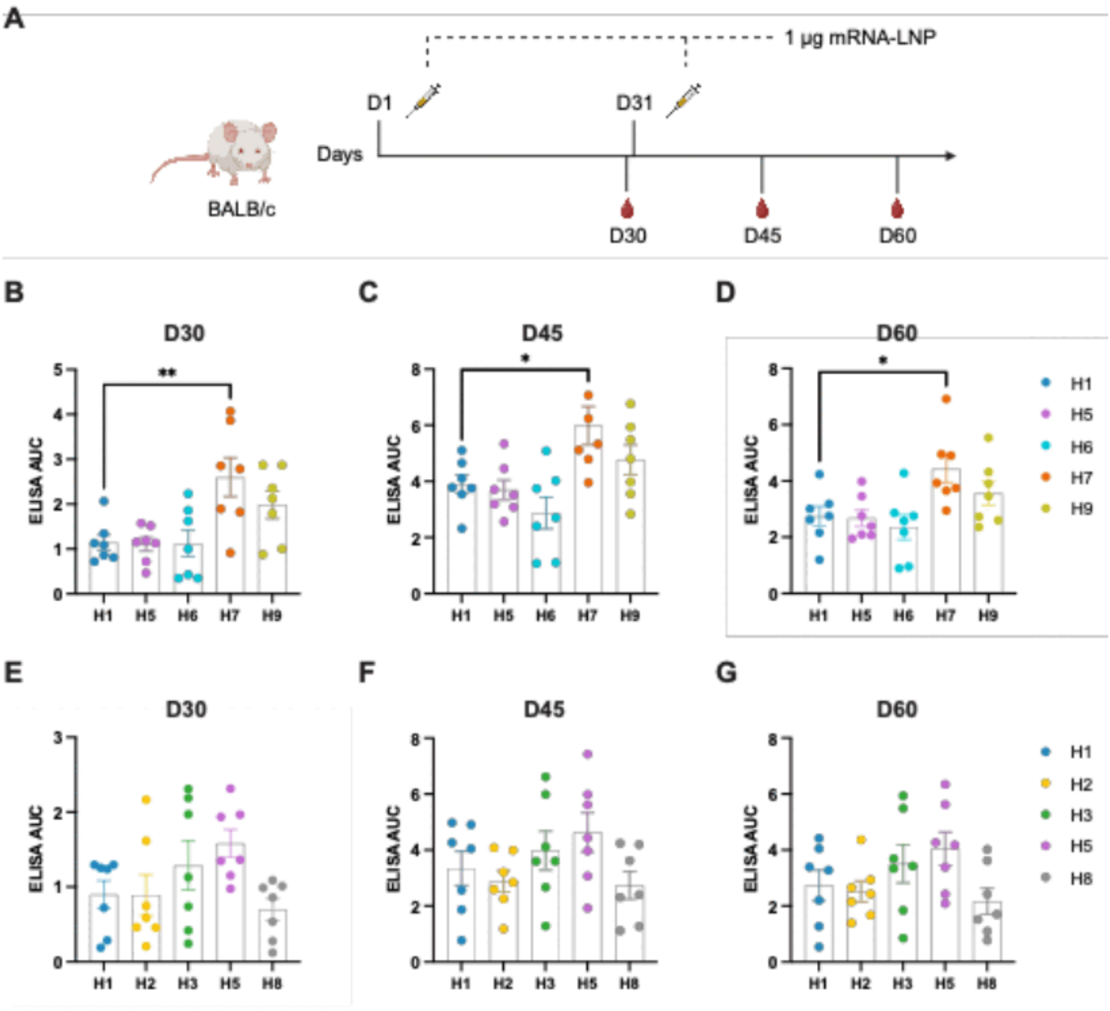

Figure 2

**An FIV late domain enhances antibody responses to a SOSIP-TM antigen.**

**A** A schedule of immunizations and blood collection. BALB/c mice were immunized intramuscularly on days 1 and 31 with 1 mg of mRNA-LNP encoding the indicated SOSIP-TM variant. Serum samples were collected on days 30, 45, and 60. Serum antibody responses measured by ELISA against the SOSIP variant 16055.ConM.8.1ds, identical to the ectodomains of each antigen. **B-D** Area under the ELISA curve (AUC) of IgG dilutions for individual mice immunized with the indicated antigens were plotted on days 30, 45, and 60, as indicated. **E-G** Experiments identical to panels B-D except that a different subset of antigens, most bearing an EPM domain, were compared. Outcomes across the five vaccine groups were compared per production batch at day 30, day 45, and day 60. For each batch and time point, a one-way ANOVA was performed and when significant, followed by Dunnett's test against the control group (H1) (**P < 0.05; **P < 0.01*).

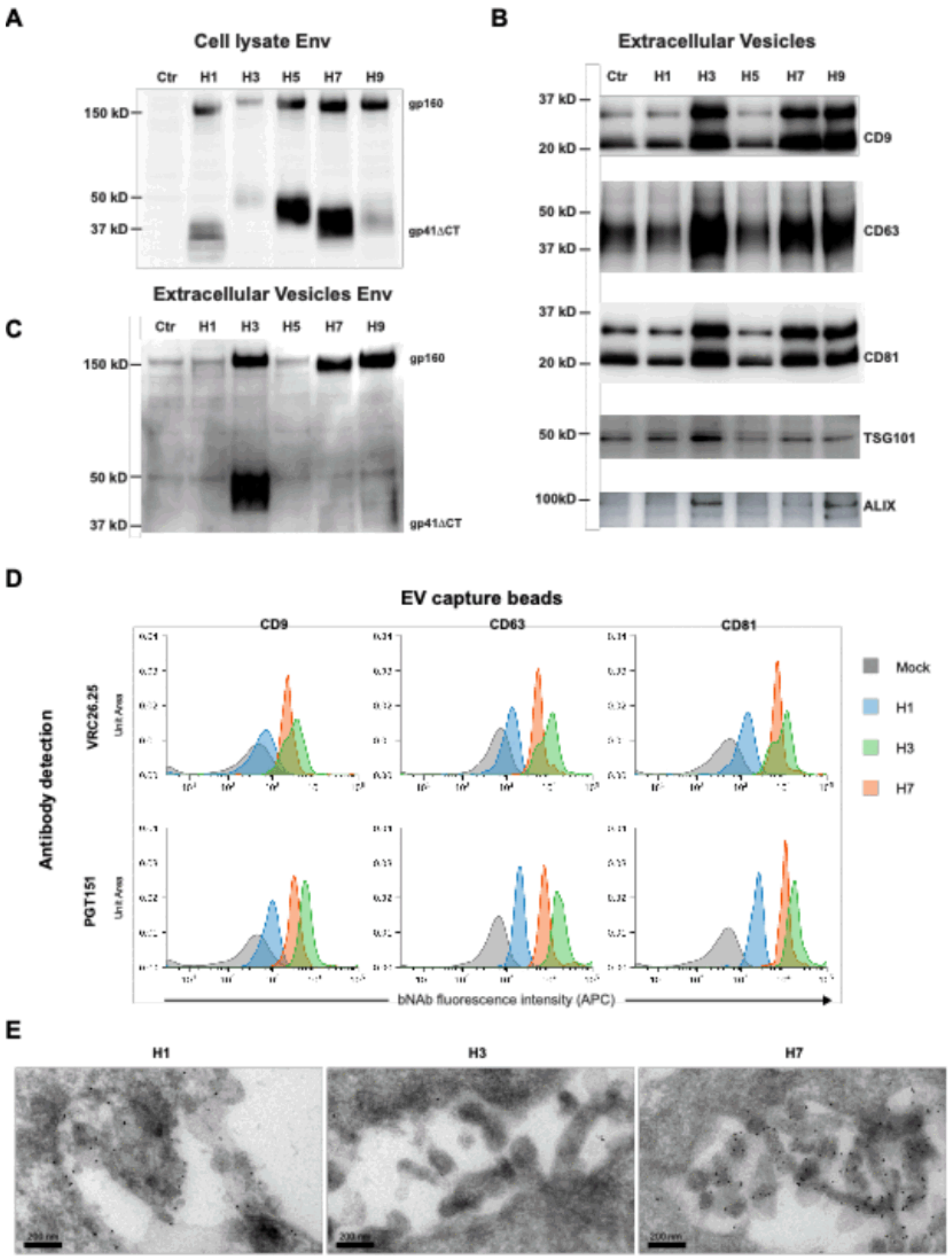

Figure 3

**Cytosolic elements SOSIP-TM antigens differentially impact cellular expression, extracellular vesicle formation and composition.**

**A** SOSIP-TM expression in cell lysates from Expi293F transduced with LNP expressing the indicated SOSIP-TM variant was monitored in a Western Blot using the anti-gp41 antibody 4E10. Note that 4E10 recognizes the gp41 component of SOSIP-TM (gp41DCT) as well as uncleaved SOSIP-TM. **B** Western blot of isolated EV probed with antibodies recognizing tetraspanin EV markers CD9, CD63, and CD81, or the ESCRT-system proteins TSG101 and ALIX. **C**. A non-reducing Western blot of isolated EV probed with the anti-gp41 antibody 4E10. Experiments in panels A-C are representative of at least two with similar results. **D** EVs from cells expressing the indicated SOSIP-TM antigen were incubated with beads conjugated to antibodies recognizing the tetraspanins CD9, CD63, and CD81, as indicated. EV-associated SOSIP-TM levels were monitored by flow cytometry using the VRC26.25 and PGT151 antibodies. **E** Representative transmission electron microscopy images of cells transfected with LNP-delivered mRNA encoding the indicated SOSIP-TM antigen. SOSIP-TM was stained with PGT151 recognized by Protein A-immunogold.

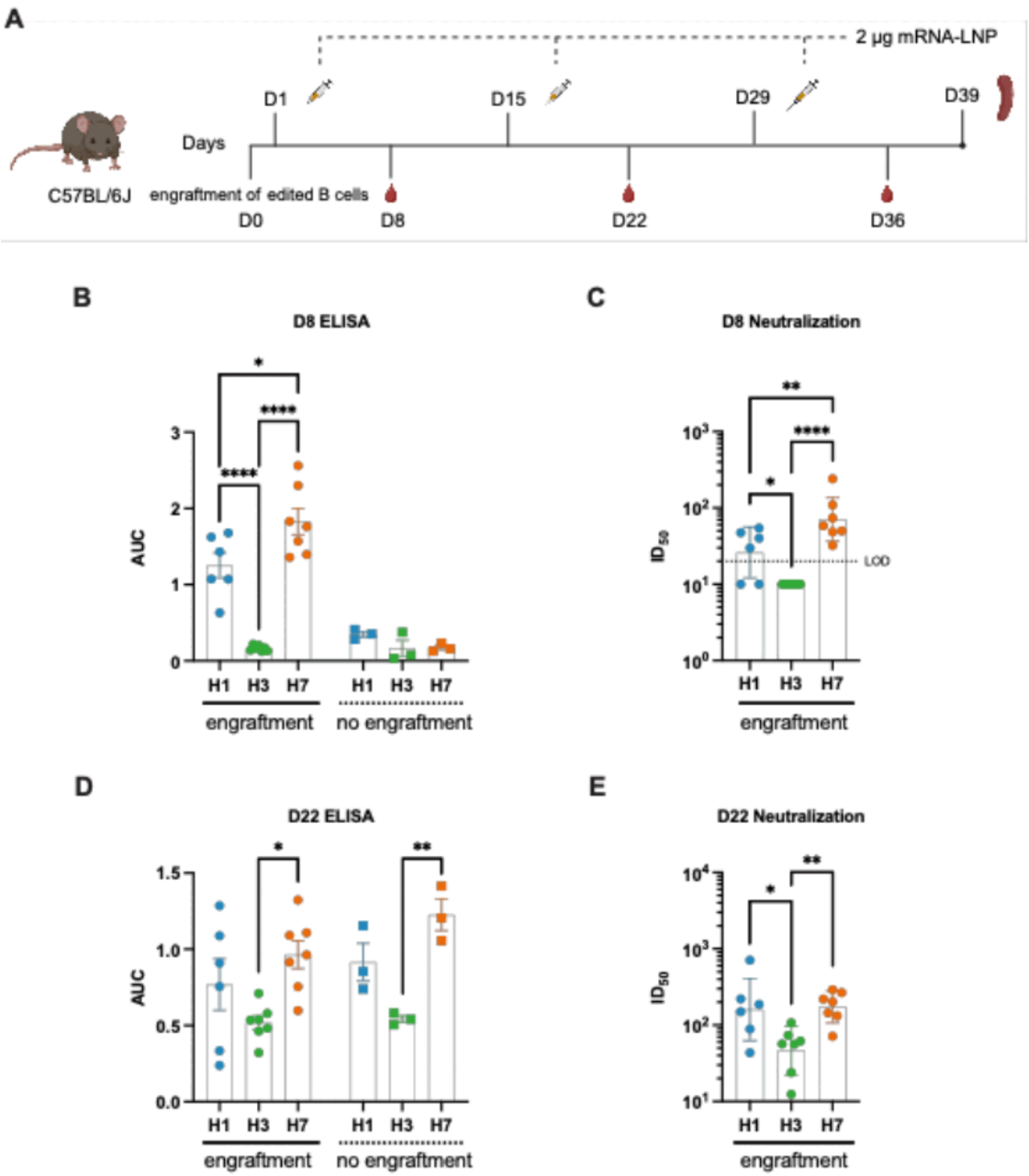

Figure 4

**An FIV late domain enhances antibody recognition of a quaternary HIV-1 Env epitope.**

**A** A schedule of immunizations and blood collections. C57BL/6 mice were engrafted with primary B cells isolated from the spleens of congenic mice engineered to express the heavy-chain CDR3 region of the HIV-1 neutralizing antibody VRC26.25, recognizing a quaternary apex epitope of SOSIP-TM. Mice so engrafted as well as unengrafted controls were immunized intramuscularly 1, 15, and 29 days post engraftment with 2 mg of mRNA-LNP encoding the indicated SOSIP-TM variant. Serum samples were collected on days 8, 22, and 36. **B,D** Serum antibody responses measured by ELISA against the SOSIP variant 16055.ConM.8.1ds. The AUC of IgG titers for individual mice were plotted on days 8 (panel B) and 22 (panel D), as indicated. Each dot represents the AUC measured from serum from an individual mouse. **C,E** Neutralization potencies of sera isolated at days 8 (panel C) and 22 (panel E) against lentiviral pseudoviruses expressing the HIV-1 envelope glycoprotein (16055 isolate). Each dot represents the 50% inhibitory dilution of a single mouse. AUC values are presented as mean ± SEM and $ID_{50}$ values are presented as geometric mean ± geometric SD. Statistical significance was determined using a one-way ANOVA, and when significant, followed by Tukey's post hoc test (*$P < 0.05$; **$P < 0.01$; ***$P < 0.001$; ****$P < 0.0001$*).

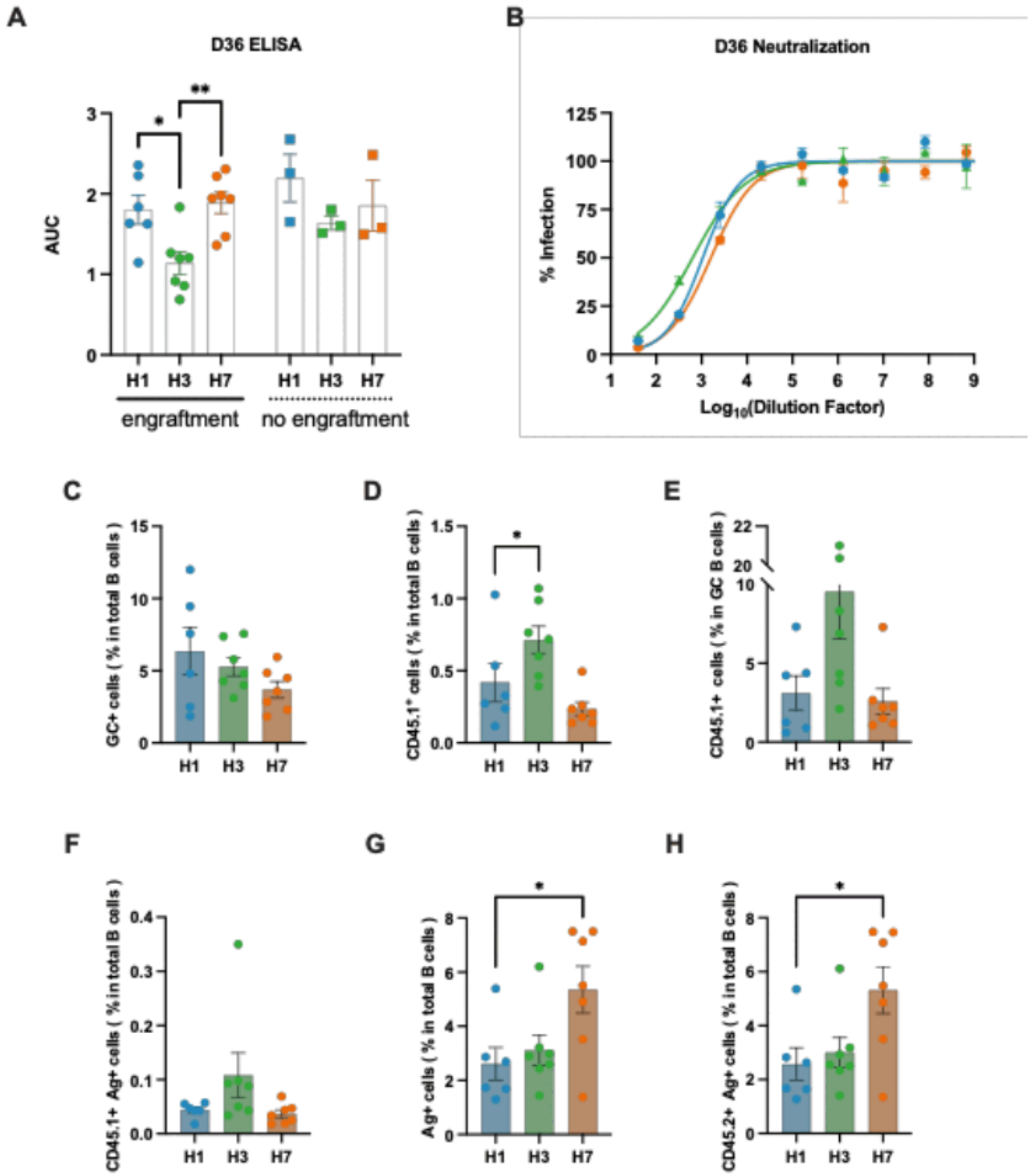

Figure 5

**Germinal center responses to SOSIP-TM variants after a third immunization.**

**A** Serum antibody responses were measured as in Fig. 4 by ELISA against the SOSIP variant 16055.ConM.8.1ds. The AUC of IgG dilutions for individual mice were plotted for day 36. **B** Neutralization of the HIV-1 isolate 16055 by sera harvested from mice engrafted with B cells expressing the VRC26.25 HCDR3 after each of three immunizations with H1, H3, and H7 SOSIP-TM variants are presented. **C-H**. Comparison of spleen-B cells in these groups of mice harvested at day 39 after the third immunization. The proportion of germinal center (GL7+, CD38-) cells of total B cells, engrafted (CD45.1+) cells of total B cells, engrafted cells of germinal center B cells, engrafted and antigen-positive cells of total B cells, antigen-positive of total B cells, and antigen-positive host (CD45.2+) cells of total B cells is shown. The cell subpopulation counts were modeled beta-binomial regression in generalized regression where vaccine group was the fixed effect. When the main test p value <0.05, Dunnett's test against the control group (H1) was performed.

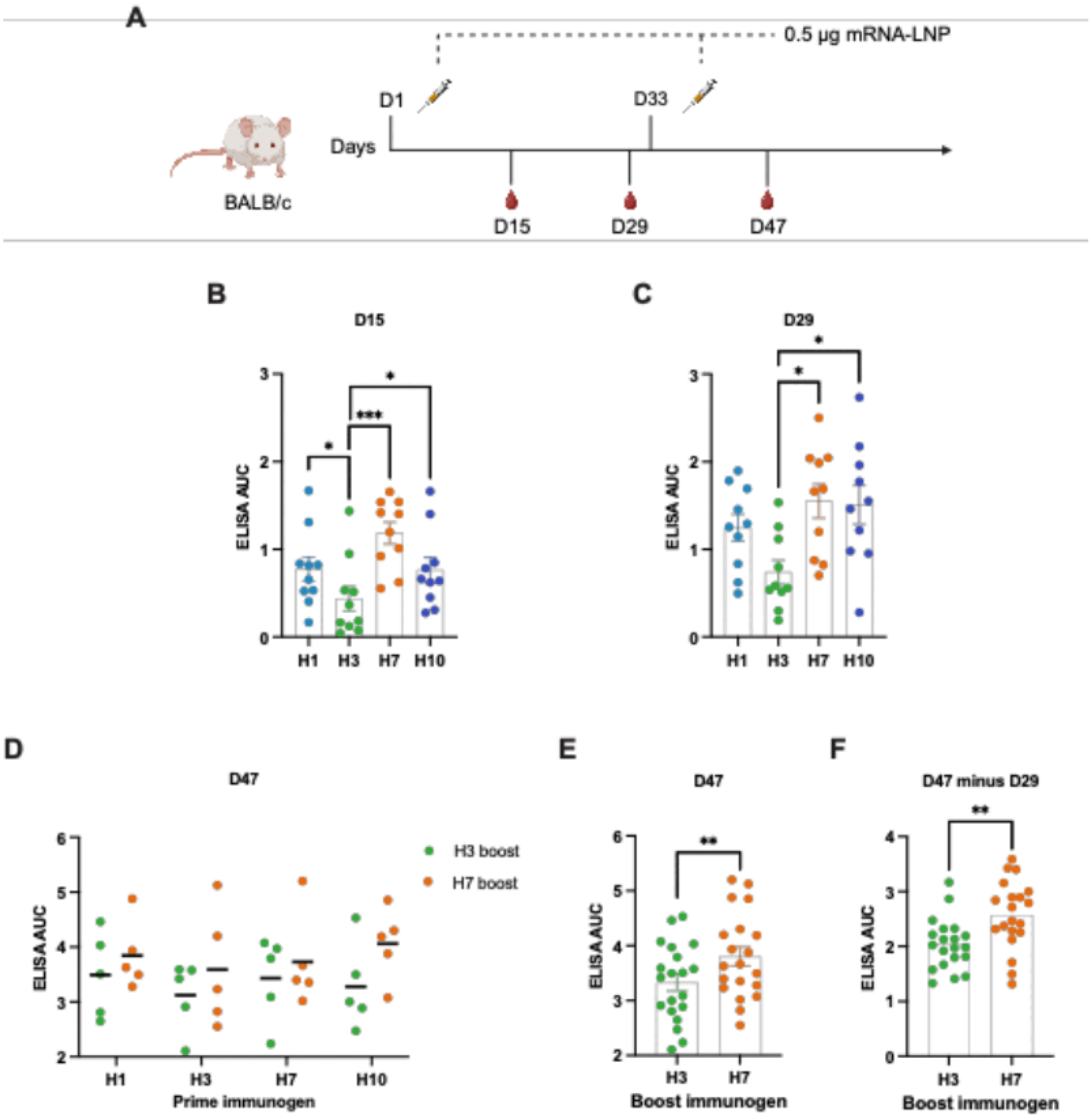

Figure 6

**FIV-p2 domain promotes immunogenicity of SOSIP-TM for both prime and boost immunizations.**

**A** A schedule of immunizations and blood collection. BALB/c were immunized intramuscularly at days 1 and 33 with 0.5 mg of mRNA-LNP encoding the indicated SOSIP-TM variant. **B-C** ELISA studies similar to those in Fig. 2, except that H1, H3, H7 and a new SOSIP-TM variant, H10, with two tamden FIV p2 domains fused to its cytoplasmic domain, are presented. Sera from day 15 (panel B) and day 29 (panel C) reflect responses to a single prime vaccination. **D** Each group of 10 in panels C were divided into two groups of five with roughly equivalent serum responses and immunized at day 33 with either H3 or H7 antigens, as shown. Blood was collected 18 days later and analyzed as in panels B and C. **E-F** Each boost group was combined as indicated, and compared for total ELISA post-boost response, for the net gain in response between the boost (day 47 sera) and prime (day 29 sera) immunization. Post-boost ELISA AUC values were analyzed by ANCOVA, with prime construct, boost construct, and their interaction as fixed effects and prime-stage ELISA AUC as a covariate. Adjusted responses are reported as least-squares means; two-sided $P < 0.05$ was considered significant.

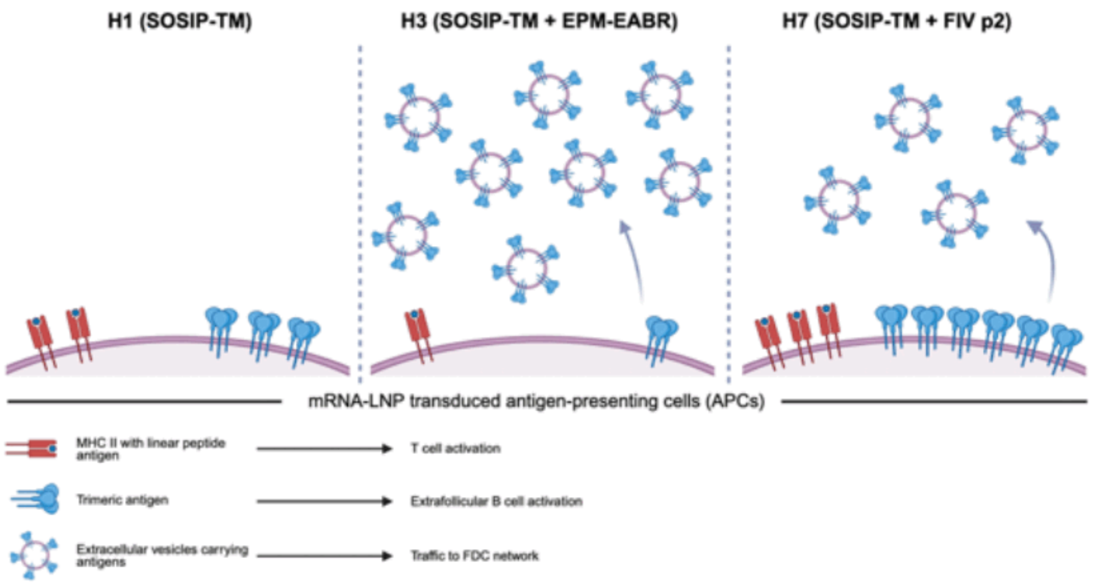

Figure 7

**Differences among H1 (unmodified), H3 (EPM-EABR), and H7 (FIV p2) SOSIP-TM antigens presented on an mRNA LNP-transfected antigen presenting cell.**

Each of the three SOSIP-TM antigens may be recognized differently by B and T cells, based on their total expression, which can impact MHC II-presentation to helper and regulatory T cells, cell-surface expression of folded antigens, which can directly engage cognate B cells at the extrafollicular B- and T-cell zone interface, or EV secretion, which can increase the total antigen in the B-cell follicles. Here we suggest that high expression of folded antigen on the surface of LNP-mRNA-transfected cells, a property of H7, can impact the generation of early extrafollicular plasma cells, resulting in higher initial serum titers. These higher titers promote greater responses to the antigen after a boost, but can also blockade specific epitopes recognized in the early response. FIV p2 also promotes EV formation, which may contribute further to these boost responses. H3, which results in lower cellular expression but higher EV formation, raised lower serum titers after early vaccinations, implying a role for antigen anchored at membrane of LNP-transfected cells.

### Supplementary Files

This is a list of supplementary files associated with this preprint. Click to download.

- UncroppedGelsforLietal.pdf
